# Research progress on the use of polyphenols in the treatment of Crohn’s disease

**DOI:** 10.3389/fphar.2025.1704901

**Published:** 2025-11-20

**Authors:** Xianmei Zhong, Qian Dai, Jing Li, Jie Yang, Xi Zheng

**Affiliations:** 1 Department of Pharmacy, Personalized Drug Research and Therapy Key Laboratory of Sichuan Province, Sichuan Provincial People’s Hospital, School of Medicine, University of Electronic Science and Technology of China, Chengdu, China; 2 Department of Pharmacy, Nanbu People’s Hospital, Nanbu, China; 3 Department of Pharmacy, The Fourth Affiliated Hospital of Southwest Medical University, MeiShan, China; 4 Sichuan Provincial People’s Hospital Medical Group Sichuan Investment Xichang Hospital, Xichang, China

**Keywords:** polyphenols, crohn’s disease, anti-inflammatory, antioxidant, inflammatory bowel disease

## Abstract

Crohn’s disease (CD) is a chronic inflammatory bowel disease (IBD), and its specific etiology is still unclear. However, it is currently widely believed that this disease is the result of interactions among four main factors: host genetic factors, environmental factors, dysbiosis of the gut microbiota, and an abnormal immune system response. In recent years, polyphenolic compounds have become a research hotspot due to their anti-inflammatory, antioxidant, and microbial regulatory properties, demonstrating potential application value in the treatment of CD. This review aimed to explore the mechanism of action of polyphenolic compounds in the treatment of CD, as well as their progress in animal model research and clinical practice.

## 1 Introduction

CD is an IBD that can affect any part of the digestive tract, from the mouth to the anus, but the most common areas are the small intestine and colon (Ballester Ferré et al., 2018). There are regional differences in the global distribution of this disease. Its incidence rate is higher in North America and Western Europe and lower in Asia and Africa. However, in recent decades, the incidence rate of CD in Asian and African countries has increased (Ng et al., 2017). With the improvement of disease awareness and the progress of diagnostic technology, more CD patients have been diagnosed, which may also explain the increase in incidence and prevalence.

CD can occur at any age, but it is most commonly diagnosed among young people (Kalla et al., 2014). The initial symptoms of CD mainly include abdominal pain, diarrhea, weight loss, fatigue, fever, chills, nausea, vomiting, and bloody stools. It should be noted that the symptoms of CD vary from person to person and may change over time (Feuerstein and Cheifetz, 2017). There is currently no cure for CD, and the treatment strategy mainly involves controlling inflammation, preventing complications, and improving patient quality of life. The current common treatment methods include drug therapy (anti-inflammatory drugs, corticosteroids, immunomodulators, biologics, small molecule drugs), nutritional support, surgical treatment, symptomatic treatment, and lifestyle adjustments (Gomollón et al., 2017). Treatment methods need to be personalized based on the patient’s specific condition, drug response, and personal preferences. Therefore, new treatment strategies should be sought to provide patients with additional treatment options.

Polyphenols are a class of compounds that are widely present in plants. Polyphenols are known for their powerful antioxidant properties. They can capture free radicals, thereby reducing oxidative stress and preventing cell damage. Polyphenols can form chelates with metal ions such as iron and copper, which helps prevent the generation of free radicals catalyzed by metals. Polyphenols also have anti-inflammatory effects and can inhibit the release and synthesis of inflammatory mediators. Moreover, certain polyphenols have antibacterial properties and can inhibit or kill pathogens, including bacteria, viruses, and fungi (Zhang et al., 2021). Currently, studies in clinical practice have shown that polyphenols can interfere with the growth and spread of cancer cells, exerting their anticancer potential (Wang et al., 2022). Meanwhile, polyphenols have been shown to have protective effects on the cardiovascular system (Iqbal et al., 2023).

## 2 Overview of polyphenol compounds

**FIGURE 1:**
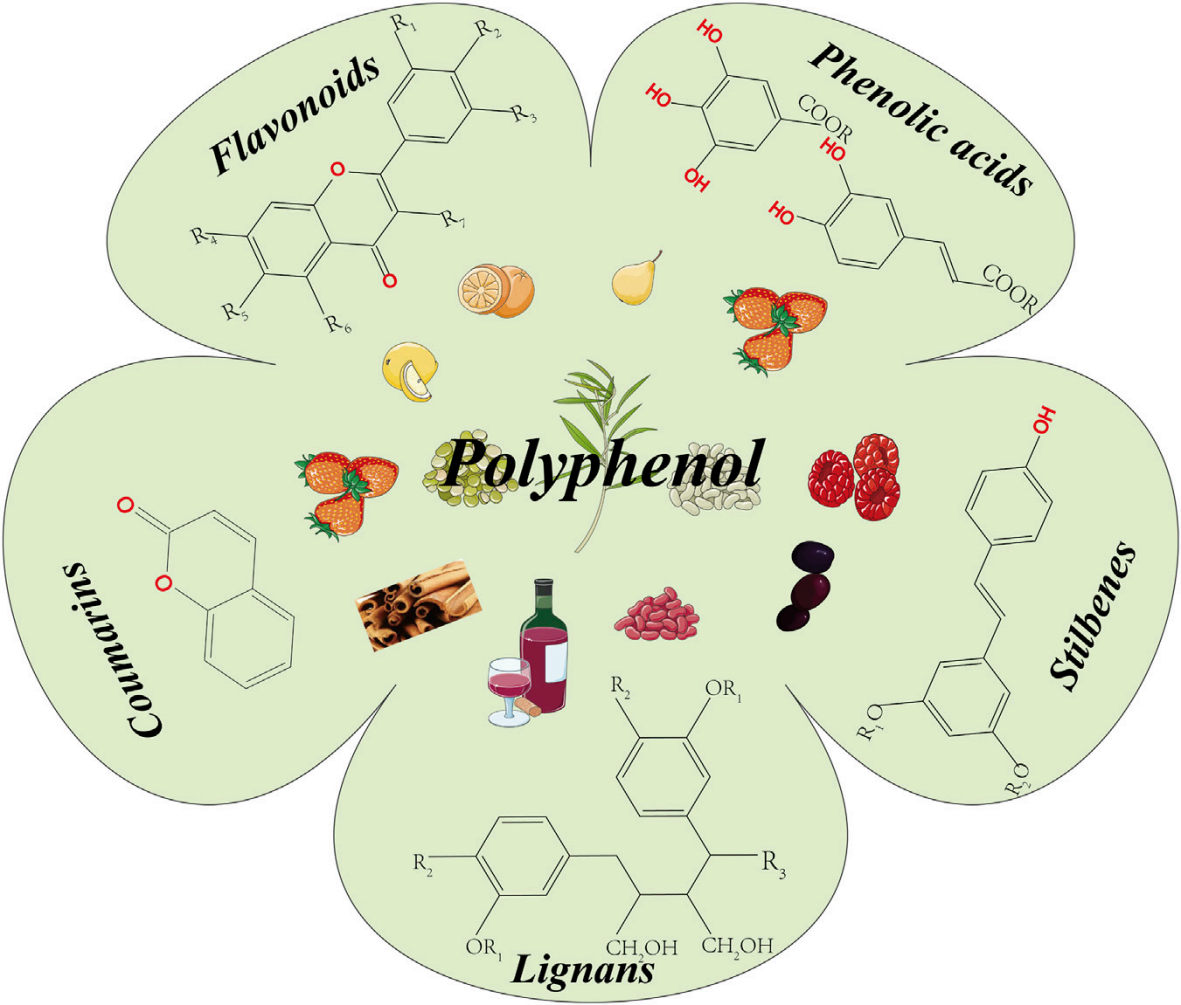
Classification of polyphenols. Flavonoids, phenolic acids, stilbenes, lignans and curvuminoids.

The structure of polyphenolic compounds is characterized by the composition of multiple phenolic structural units. Polyphenols are widely distributed in every part of plants, and fruits, vegetables, tea, coffee beans, nuts, seeds, bark, and flowers all contain abundant polyphenols (Fu et al., 2011). According to their chemical structure, polyphenols can be roughly divided into five categories. The first type is flavonoids (C6-C3-C6). The second type is phenolic acids (C6-C1/C6-C3). The third type is stilbenes (C6-C2-C6). The fourth type is lignans (C6-C3-C3-C6). The fifth type is coumarins (C6-C3-C1-C3-C6) (Figueira et al., 2017) (Figure 1).

The largest class of phenolic compounds in plants is flavonoids, for which more than 10,000 different structures have been identified (Cheynier et al., 2013). Flavonoids are heterocycles containing two benzene rings and one heterocycle containing three carbon atoms. These compounds can be further divided into six subcategories: flavanols, flavanones, flavones, isoflavones, anthocyanins, and flavonols (Di Lorenzo et al., 2021). Flavonoids are widely distributed in foods such as tea, cocoa, apples, grapes, and berries and are an important subclass of flavonoids (Manach et al., 2004). Flavanols mainly include catechins, gallocatechins, catechin 3-gallate, gallocatechin 3-gallate, epicatechin 3-gallate, and epicatechin 3-gallate (Zhuang et al., 2023). The related flavanols also include flavanones, whose main representative compounds include hesperidin, naringin, hesperidin, and naringin (Santhiravel et al., 2022). In addition to flavanol compounds, isoflavones are called phytoestrogens or phytoketones due to their structural similarity to estrogen. These chemical compounds mainly come from legumes and are the main source of daidzein, genistein, and daidzein (Křížová et al., 2019). Anthocyanins, mainly as compounds that give color to flowers and fruits, also exist in plants in the form of glycosides, which are connected with different types of sugars to form stable compounds. The anthocyanin compounds included cyanidin, delphinidin, malvidin, peonidin, petunidin, and pelargonidin (Mattioli et al., 2020). Flavonol ketone compounds are complex phenolic compounds composed of flavanols and lignin and are represented by silymarin, which is present in the fruit of Silybum marianum (L.) Gaertn (Valentová et al., 2020).

Phenolic acids are mainly divided into two categories: hydroxybenzoic acid derivatives and hydroxycinnamic acid derivatives (Khadem and Marles, 2010). Hydroxybenzoic acid derivatives possess the basic framework of benzoic acid, and their representative components include gallic acid, vanillic acid, para hydroxybenzoic acid, salicylic acid, and dehydrotarbic acid (da Silva et al., 2023). On the other hand, hydroxycinnamic acid derivatives are based on cinnamic acid as the core structure, which results in the formation of components such as caffeic acid, ferulic acid, p-coumaric acid, and sinapic acid (Alam et al., 2016). These compounds are more common in coffee, oats, and green tea (El-Seedi et al., 2012).

Stilbene compounds have a specific structure in which two benzene rings are connected through an ethylene bridge. Among the numerous stilbene compounds, resveratrol, piceatannol, pterostilbene, and 2,3,5,4′-tetrahydroxystilbene-2-O-β-D-glucoside are particularly well-known (Teka et al., 2022). Resveratrol is the most extensively studied compound and is found mainly in grape skins, red wine, peanuts, and certain berries (Al-Khayri et al., 2023).

The basic structure of lignans consists of two or more phenylpropane units (such as cinnamic acid, cinnamyl alcohol, propenylbenzene, and allylbenzene). Based on the linkage pattern of these monomer units, lignans are classified into two categories: those formed by monomers linked at the β-β′ position are termed “classical lignans”; conversely, compounds where the main structural units are coupled in any other manner are grouped as “neolignans”. To date, over 200 classical lignans and more than 100 neolignans have been isolated and characterized from the plant kingdom. They primarily exist as dimers, though some occur as trimers or tetramers. In plants, lignans are mostly found in their free form, while some can bind with glycosyl groups to form derivatives like glycosides. Research indicates that lignans possess biological activities including antitumor, antioxidant, and antimicrobial properties (Cui et al., 2020).

Coumarin is a series of compounds containing a coumarin ring structure that is composed of a benzene ring and a pyran ring connected by carbon atoms (Sharifi-Rad et al., 2021). Coumarins are divided into four categories: pure coumarins, furan coumarins, pyranocoumarins, and pyranosubstituted coumarins (Küpeli et al., 2020). More than 1,300 types of coumarins have been identified from plant sources. Currently, research has shown that coumarins have pharmacological activities, such as anti-inflammatory, anticoagulant, antibacterial, antiviral, anticancer, antihypertensive, antituberculosis, anticonvulsant, antilipogenic, and antihyperglycemic effects (Sharifi-Rad et al., 2021).

In our diet, polyphenolic compounds not only add flavor to food and provide color but also have many potential health benefits. Understanding the characteristics and mechanisms of various polyphenols can provide a scientific basis for the development of new therapies and health products.

## 3 The pathogenesis of CD

**FIGURE 2:**
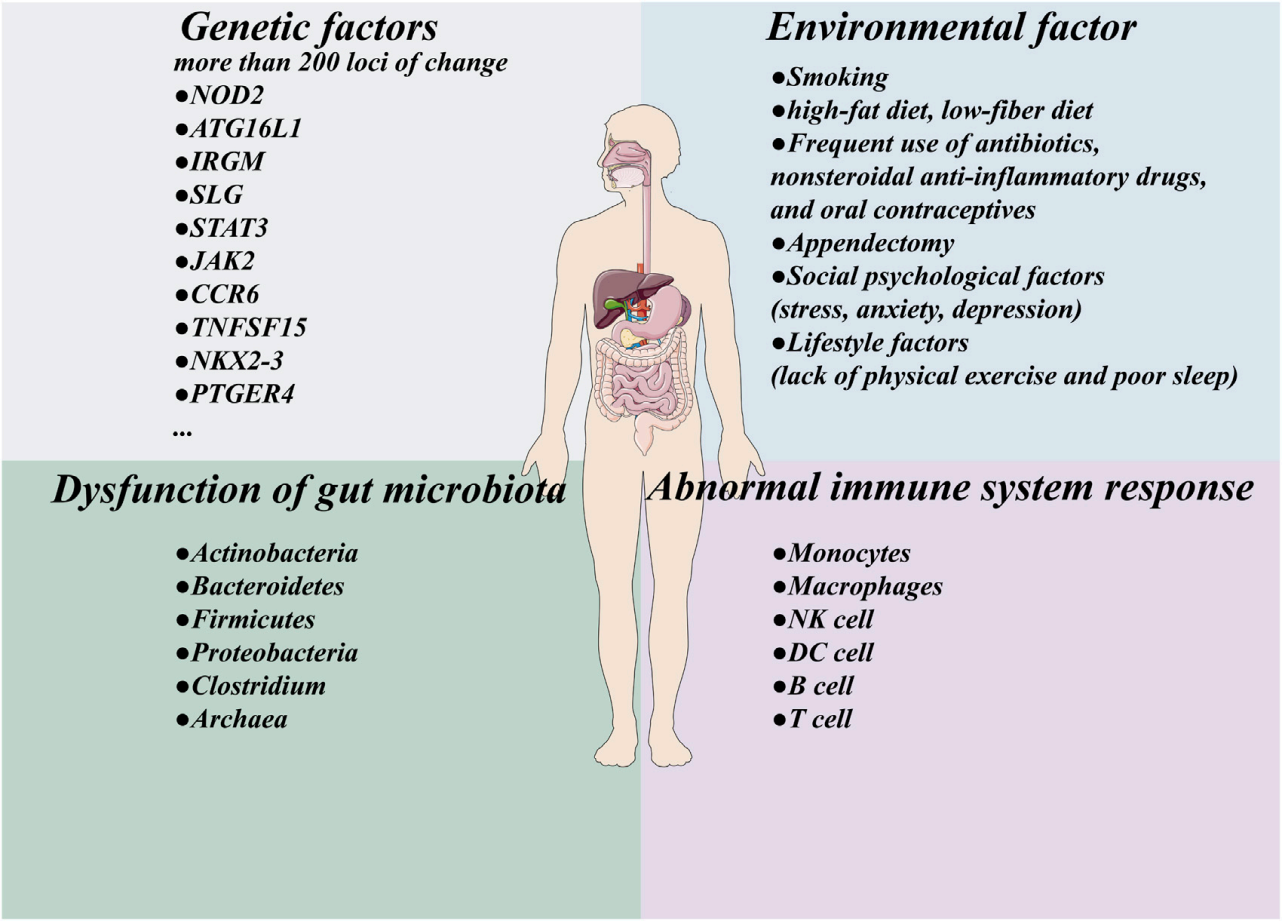
Pathogenesis of Crohn’s disease. host genetic factors, environmental factors, dysbiosis of the intestinal microbiota, and an abnormal immune system response.

CD is a chronic inflammatory disorder of the gastrointestinal tract. Its clinical course is often severe; a meta-analysis indicates that the cumulative 5-year hospitalization risk for CD patients is as high as 44.3% (Tsai et al., 2022). Furthermore, approximately 40%–50% of patients require major surgical intervention within 10 years of diagnosis (Cho et al., 2022). Currently, the precise pathogenesis of CD remains incompletely understood. However, it is widely accepted that the disease arises from the complex interplay of four core factors: host genetic susceptibility, environmental factors, gut microbiota dysbiosis, and aberrant immune system responses (Figure 2).

### 3.1 Genetic factors

Genetic components play a pivotal role in the pathophysiology of CD. Genome-wide association studies have identified over 200 genetic loci associated with CD susceptibility. Notably, individuals with a family history of CD exhibit a significantly elevated disease risk (Wang and Picco, 2017; Sazonovs et al., 2022). These susceptibility loci do not act in isolation but converge on core biological pathways governing intestinal homeostasis, including: (1) Autophagy impairment: Mutations in key autophagy-related genes (e.g., ATG16L1, IRGM, and MST1) disrupt cellular autophagy, compromise intracellular bacterial clearance, and may perpetuate inflammatory responses (Gabbani et al., 2017; Mehto et al., 2019). (2) Intestinal epithelial barrier dysfunction: Genetic variants in genes such as PTPN2 and PCDH20 are implicated in the structural and functional integrity of the mucosal physical barrier (Scharl et al., 2009; Huang et al., 2023). (3) Impaired Innate Immune Recognition and Bacterial Sensing: Genetic variants in NOD2 (recognizing bacterial cell wall components), ITLN1 (detecting galactofuranosyl residues in microbial cell walls), and CARD9 compromise Paneth cell antimicrobial responses and the host’s ability to discriminate between commensal and pathogenic microbes. This results in defective bacterial clearance and aberrant immune activation (Gabbani et al., 2017; Sidiq et al., 2016; Wang et al., 2010; Janse et al., 2011; Singh et al., 2006; Liu and Stappenbeck, 2016). (4) Dysregulated Adaptive Immunity (T helper 17 [Th17] Pathway) and Cytokine Signaling Abnormalities: Functional aberrations in genes such as IL23R, STAT3, JAK2, IL12B, CCR6, and TNFSF15 directly disrupt Th17 cell differentiation, function, and Th17-mediated immune responses (Gabbani et al., 2017; Prager et al., 2012; Brand, 2009). Concurrently, variants at loci such as PTPN2, PTPN22, and STAT3 impair negative regulation of cytokine signaling, exacerbating inflammatory responses and apoptosis (Prager et al., 2012; Prager et al., 2014; Li et al., 2015; Sharp et al., 2018). (5) Other Immune Regulation and Positioning Defects: Loci including NKX2-3 (upregulated in B cells and intestinal tissues, potentially affecting lymphocyte homing) and the 5p13 chromosomal region (harboring genes such as PTGER4, which influences barrier function and inflammatory responses) also contribute to the genetic susceptibility to CD (Gabbani et al., 2017; Prager et al., 2012; Yu et al., 2009).

### 3.2 Environmental factors

The interplay between environmental factors and genetic susceptibility collectively influences the risk and disease course of CD. Smoking is a well-established risk factor that may significantly elevate disease risk by modulating immune responses, intestinal mucosal function, and gut lavage (Berkowitz et al., 2018). Modern Westernized dietary patterns (typically characterized by high fat, high sugar, processed food consumption, and low dietary fiber intake) demonstrate a significant association with increased CD risk (Altajar and Moss, 2020). Studies have revealed that high-fat diet intake correlates with elevated CD risk (Hou et al., 2011), while high-protein diets may alter gut microbiota composition by reducing the abundance of beneficial bacteria (e.g., Roseburia/Eubacterium rectale), thereby diminishing butyrate production (Altajar and Moss, 2020). Conversely, high-fiber diets are associated with reduced CD risk, with their protective effects attributed to promoting beneficial microbial growth and enhancing short-chain fatty acid (e.g., butyrate) production, which exhibits anti-inflammatory properties and maintains intestinal epithelial barrier integrity (Altajar and Moss, 2020; Hou et al., 2011). Furthermore, frequent use of antibiotics, non-steroidal anti-inflammatory drugs (NSAIDs), and oral contraceptives may contribute to increased disease risk, suggesting potential mechanisms involving gut microbiota dysbiosis (Narula et al., 2023). Some studies indicate that young individuals subjected to appendectomy within the initial postoperative years exhibit the highest CD risk. Appendectomy-induced alterations in gut microbiota may influence inflammatory bowel disease pathogenesis (Fantodji et al., 2022). Psychosocial factors (e.g., stress, anxiety, and depression) and lifestyle factors (e.g., physical inactivity and poor sleep quality) are also recognized as potential risks and have been implicated in CD development (Neal et al., 2022). Notably, low vitamin D levels in CD patients correlate with disease activity, and vitamin D supplementation appears beneficial for improving clinical scores and reducing inflammation (Basson, 2014; White, 2018). Comprehensive understanding and management of these environmental factors are crucial for CD prevention and treatment.

### 3.3 Dysbiosis of the intestinal microbiota

The dysbiosis of gut microbiota in CD patients is a key factor affecting disease development and manifests as a decrease in beneficial bacteria and an increase in potential pathogenic bacteria. This disruption of the microbial community can lead to excessive activation of the immune system and intestinal inflammation. The specific manifestations of CD patients are a relative increase or decrease in the abundance of Actinobacteria, Bacteroidetes, Firmicutes, Proteobacteria, *Clostridium*, and Archaea taxa (Caparrós et al., 2021). The abundance of the beneficial genus Bifidobacterium in the healthy gut is reduced in CD patients (Favier et al., 1997). A study revealed that the abundance of bacterial species that produce butyrate in the fecal microbiota of CD patients decreased, with significant reductions in the abundances of genera such as Bacteroidetes, Eubacterium, Fecal *Bacillus*, and Ruminococcus (Takahashi et al., 2016). The reduction of this butyrate producing bacterium leads to a decrease in the production of key short chain fatty acids, especially butyrate (Takahashi et al., 2016). Butyrate has anti-inflammatory effects and can inhibit the release of the inflammatory factor interleukin-6 (IL-6) and lipopolysaccharides (LPS)-induced tumor necrosis factor-α (TNF-α) release and activate the TNF-α-mediated inhibition of the nuclear factor kappa-B (NF-κB) inflammatory pathway (Tedelind et al., 2007). Therefore, the reduction in butyrate production is a key functional defect in CD, exacerbating intestinal inflammation. In addition, the presence of adhesive invasive *Escherichia coli* is closely related to severe mucosal microbiota dysbiosis in CD patients and may hinder the colonization of beneficial bacteria (Zhilu et al., 2021). Notably, the changes in the number of methane-producing archaea in patients with CD are also worth investigating (Ghavami et al., 2018). At present, there is relatively little research on the gut virus community, but there is evidence to suggest that the dynamics of bacterial and viral (mainly bacteriophage) communities in fecal samples from CD patients undergo changes (Rashed et al., 2022). Antimicrobial peptides are essential components of the host defense system and play a regulatory role in the composition of the gut microbiota. In patients with CD, the expression of AMPs—such as α-defensins (e.g., HD5, HD6), calprotectin (CRP), lactoferrin, lysozyme, elafin, galectin-1, galectin-3, cathelicidin, hepcidin, and lipocalin—is frequently dysregulated (Gubatan et al., 2021). Notably, the reduction in α-defensins HD5 and HD6 impairs their bactericidal activity and barrier-protective functions, thereby compromising intestinal homeostasis (Coretti et al., 2017).

### 3.4 Abnormal immune system response

In CD patients, impaired intestinal epithelial barrier function allows luminal contents to breach the mucosal barrier, leading to the sustained activation of immune cells (D et al., 2015). Activated monocytes, macrophages, and dendritic cells (DCs) excessively produce reactive oxygen species (ROS) and secrete key cytokines. Among these, core driver cytokines—including interleukin-23 (IL-23), interleukin-12 (IL-12), interleukin-18 (IL-18), and transforming growth factor-β (TGF-β)—promote the differentiation of naïve T cells into Th17 and T helper 1 (Th1) cells (Kitahora et al., 1988; Uhlig and Powrie, 2018). Abnormally activated Th17 and Th1 cells secrete large amounts of pro-inflammatory cytokines such as interleukin-17 (IL-17), interferon-γ (IFN-γ), and TNF-α. These interact with activated tissue-resident macrophages, forming a self-sustaining inflammatory amplification loop (Uhlig and Powrie, 2018). The IL-23/Th17 axis occupies a central position in CD pathogenesis. IL-23 promotes the expansion of differentiated Th17 cell populations by acting on cells expressing the IL-23 receptor (IL-23R). Th17 cells activate inflammatory cascades via Janus kinase (JAK) and signal transducer and activator of transcription (STAT)-mediated pathways, which may trigger or exacerbate inflammation (Shen and Durum, 2010). Consequently, the IL-23/Th17 axis has emerged as one of the premier therapeutic targets (e.g., IL-23p19 inhibitors) (Fanizza et al., 2023). Simultaneously, activated natural killer (NK) cells secrete pro-inflammatory cytokines and chemokines (e.g., C-C motif chemokine receptor 2 [CCR2], regulated on activation, normal T-Cell expression and secretion [RANTES]), further killing severely functionally impaired regulatory T cells (Tregs) and activating effector T cells (Samarani et al., 2020). The defective inflammatory suppressive function of Tregs is a core element of CD immune dysregulation and an important therapeutic target (Clough et al., 2020). Furthermore, B cells exhibit features of chronic activation—including granulomatous localization and increased IgA/IgG maturity—which contribute to chronic inflammation. While significant in driving CD inflammation, their role is generally considered secondary to the hyperactivated T cells (Th1/Th17) and the macrophage/monocyte system (Timmermans et al., 2016). This persistent immune activation ultimately leads to intestinal wall edema, ulceration, stricturing, fistula formation, and an increased risk of intestinal obstruction, perforation, and colorectal cancer (Ballester Ferré et al., 2018; Me et al., 2021). Therefore, CD treatment requires comprehensively repairing the barrier, modulating the microbiota, and critically focusing on targeting and correcting key immune dysregulation mechanisms such as the overactivated IL-23/Th17 axis and restoring Treg function.

## 4 The potential role of polyphenols in the treatment of CD

**FIGURE 3:**
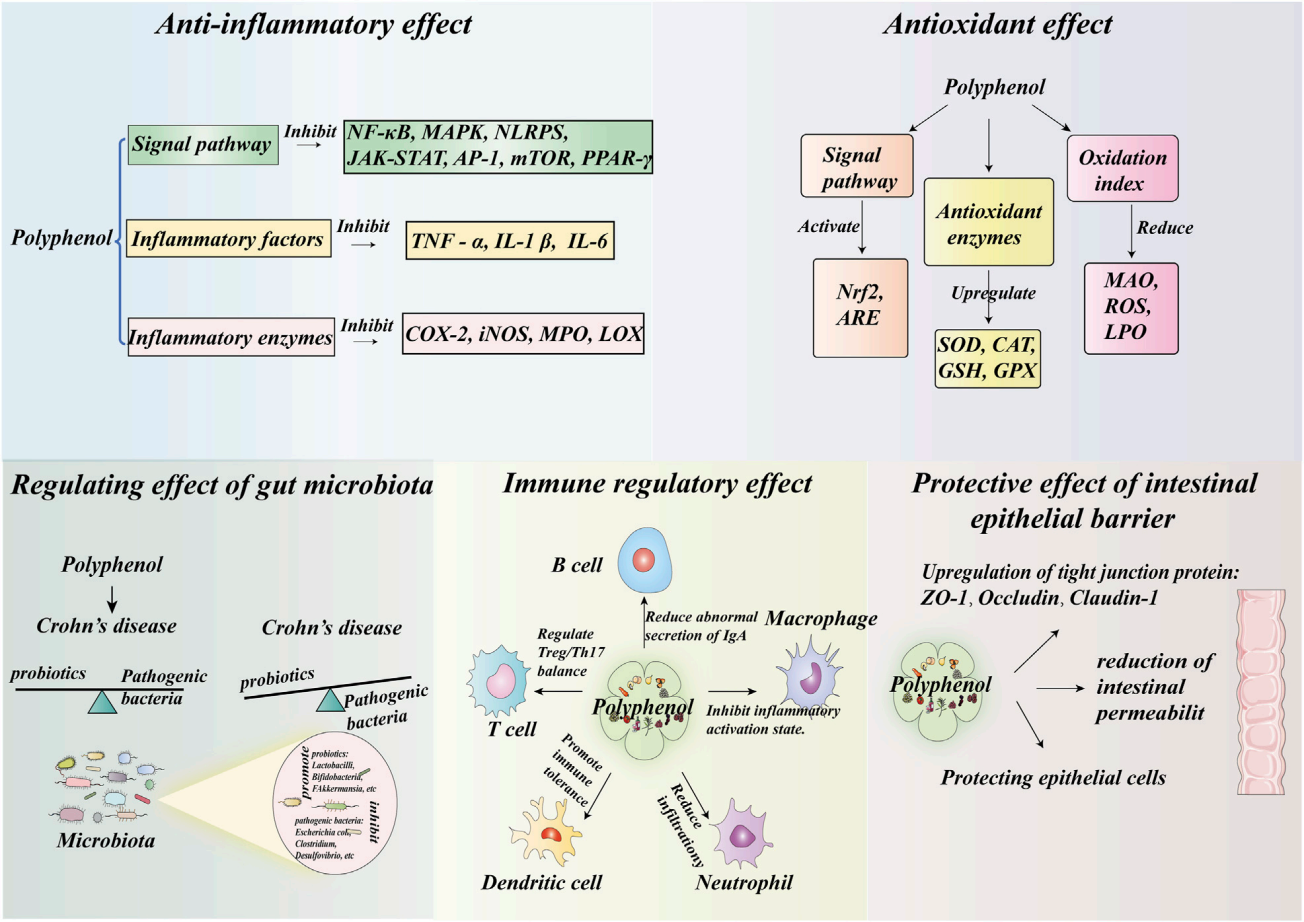
Potential Mechanisms of Polyphenol Action. (1) Anti-inflammatory Effects: Polyphenols exert multi-layered anti-inflammatory effects by targeting core anti-inflammatory signaling pathways, inhibiting inflammatory factors, and suppressing inflammatory enzymes. (2) Antioxidant Properties: Antioxidant properties: Polyphenols protect cellular components from oxidative damage by activating antioxidant signaling pathways, upregulating antioxidant enzymes, and scavenging ROS. (3) Gut Microbiota Modulation: Polyphenols modulate the gut microbiota by suppressing specific pathogens while promoting the growth and activity of beneficial bacteria. (4) Immunomodulation: Polyphenols counteract immune dysregulation through multiple mechanisms: regulating the Treg/Th17 balance, suppressing the inflammatory activation state of macrophages, reducing neutrophil and dendritic cell infiltration, and diminishing B-cell autoantibody-mediated intestinal damage. (5) Intestinal epithelial barrier protection: Polyphenols protect the intestinal epithelial barrier by upregulating tight junction proteins and reducing intestinal permeability.

Polyphenols from different categories exhibit similar mechanisms in alleviating the pathological process of CD, primarily through five aspects: anti-inflammatory effects, antioxidant activity, gut microbiota modulation, immunoregulation, and intestinal barrier protection (Table 1; Figure 3). In terms of anti-inflammatory effects, they all reduce the release of pro-inflammatory cytokines by inhibiting inflammatory signaling pathways such as NF-κB and MAPK. Regarding antioxidant activity, polyphenols enhance endogenous antioxidant enzyme activity and activate the nuclear factor erythroid 2-related factor 2 (Nrf2) pathway to bolster antioxidant capacity. In immunomodulation, various polyphenols can regulate the Treg/Th17 balance and influence macrophage polarization. With respect to gut microbial communities, they increase beneficial bacteria and reduce pathogenic bacteria. In intestinal epithelial barrier protection, they consistently upregulate tight junction protein expression and reduce intestinal permeability. However, distinct polyphenol classes also possess differential advantages: flavonoids can additionally modulate the intestinal endocrine system; phenolic acids exhibit outstanding performance in barrier repair; stilbenes, lignans, and coumarins can generate novel bioactive compounds through unique metabolic pathways. Moreover, they display specific preferences for particular bacterial species in microbiota regulation. This diversity provides a theoretical basis for combined applications.

**TABLE 1:** Potential mechanism of action of different polyphenols in the treatment of CD.

Mechanism of action	Flavonoids	Phenolic acids	Stilbenes	Lignans	Coumarins	Joint action target
Anti-inflammatory effect	Signal pathway: Inhibit NF - κ B, MAPK (ERK/JNK/p38), AP-1 Inflammatory factors: Reduce the release of TNF - α, IL-1 β, and IL-6 Inflammatory enzymes: Inhibit COX-2, iNOS, MPO activity	Signal pathway: Inhibit NF - κ B, MAPK, NLRP3 inflammasome Inflammatory factors: Reduce the release of TNF - α, IL-1 β, and IL-6 Inflammatory enzymes: Inhibit COX-2, iNOS, MPO activity Other: Ferulic acid derivatives inhibit macrophage pro-inflammatory cytokines	Signal pathways: Inhibit NF - κ B, MAPK (ERK/JNK), JAK/STAT, mTOR Inflammatory factors: Reduce the release of TNF - α and IL-1 β Inflammatory enzymes: Inhibit COX-2 and iNOS activity Other: Resveratrol inhibits p65 transcription and NEMO ubiquitination	Signal pathway: Inhibit NF - κ B, MAPK, PPAR - γ, Nrf2 Inflammatory factors: Reduce the release of TNF - α, IL-1 β, and IL-6 Inflammatory enzymes: Inhibit LOX and COX-2 activity	Signal pathway: Inhibit NF - κ B, MAPK, JAK/STAT3, NLRP3 inflammasome Inflammatory factors: Reduce the release of TNF - α and IL-1 β Inflammatory enzymes: Inhibit COX-2, 5-LOX activity Other: The combination of Qinpi Yi Su and Ophiopogon japonicus (1:1 ratio) has a synergistic anti-inflammatory effect	Signal pathway: Inhibit NF - κ B and MAPK Inflammatory factors: Reduce the release of TNF - α and IL-1 β Inflammatory; enzyme: Inhibit COX-2 activity
Antioxidant effect	Signal pathway: Activate the Nrf2/ARE pathway Antioxidant enzymes: Upregulate HO-1, SOD, CAT, GSH Oxidation index: Reduce MDA, LPO, and excessive NO.	Signal pathway: Activate Nrf2 Antioxidant enzymes: Upregulate HO-1, SOD, CAT Oxidation index: Eliminate · OH and O _2_^-^ free radicals, and reduce MDA.	Signal pathway: Activate Nrf2 Keap1 and PPAR - γ pathway Antioxidant enzymes: Upregulate HO-1, GCLC, SOD, GPx Oxidation index: Reduce MDA and eliminate ROS.	Signal pathway: Activate Nrf2 Antioxidant enzymes: Upregulate SOD, CAT, HO-1, GSH Oxidation index: Reduce MDA and eliminate ROS.	Signal pathway: Activate Nrf2 Antioxidant enzymes: Upregulate SOD, GPx, HO-1, GSH Oxidation index: Reduce MDA and eliminate ROS Other: The combination of Qinpi Yi Su and Ophiopogon (1:1 ratio) synergistically activates the Nrf2 signaling pathway	Signal pathway: Activate Nrf2 Antioxidant enzymes: Upregulate SOD, HO-1, GSH Oxidation index: Reduce MDA and eliminate ROS.
Regulating effect of gut microbiota	Beneficial bacteria: Increase lactobacilli, bifidobacteria A. muciniphila Pathogenic bacteria: Reduce *Enterococcus* and *Clostridium* Metabolites: Promote the production of SCFAs	Beneficial bacteria: Increase lactobacilli, bifidobacteria Akkermansia、Bacteroidetes Pathogenic bacteria: Reduce Enterobacteriaceae and Desulfovibrio Other: Vanillin acid enhances Firmicutes/Bacteroidetes	Beneficial bacteria: Increase lactobacilli, Akkermansia, and SCFA producing bacteria Pathogenic bacteria: Reduce *Enterococcus faecalis* Metabolic conversion: The gut microbiota metabolizes resveratrol into active derivatives such as dihydroresveratrol and puerarin	Beneficial bacteria: Increase Ruminococcus and Bifidobacterium Pathogenic bacteria: Inhibit *Clostridium* sensu stricto-1 Metabolic conversion: Glucosides (SDGs) are converted to ENL/END by *Bacteroides* and *Clostridium*	Beneficial bacteria: Increase lactobacilli and SCFA producing bacteria Pathogenic bacteria: Inhibit the formation of pathogenic bacteria (*Escherichia coli* O157: H7) biofilm Metabolic conversion: 7-methoxycoumarin is converted into coumarin acid	Increase beneficial bacteria and reduce pathogenic bacteria
Immune regulatory effect	T cells: Promote Treg differentiation and inhibit Th1/Th17 polarization Macrophages: Inhibit M1 type activation Neutrophils: Reduce infiltration DCs: Reduce infiltration	T cells: Promote Treg differentiation, inhibit Th1/Th17, balance Th1/Th2 response Macrophages: Inhibit M1 polarization Neutrophils: Reduce infiltration and migration	T cells: Inhibit Th1 and Th17, induce Treg, and induce myeloid derived suppressor cells (MDSCs) to suppress effector T cells Macrophages: Promote M2 polarization Other: Regulating immune response through upregulation of miR-101b and downregulation of miR-31	T cells: Inhibit Th17 differentiation, promote Treg proliferation, and regulate Th17/Treg balance Macrophages: Inhibit inflammatory activation state Neutrophils: reduce infiltration B cells: Reduce intestinal damage mediated by autoantibodies	T cells: Regulate Treg/Th17 balance, enhance Treg function, and reduce Th1 cell infiltration Macrophages: Inhibit M1 type pro-inflammatory response DCs: Promote the differentiation of tolerant DCs B cells: Reduce abnormal secretion of IgA	T cells: Regulate Treg/Th17 balance Macrophages: Inhibit inflammatory activation state
Protective effect of intestinal epithelial barrier	Tight junction proteins: Upregulate ZO-1, Occludin, Claudin-1 Permeability: Reduce intestinal permeability and decrease bacterial translocation Other: Promote the secretion of intestinal hormones such as glucagon like peptide-1 (GLP-1), GLP-1, and cholecystokinin (CCK), indirectly enhancing anti-inflammatory and intestinal repair effects	Tight junction proteins: Upregulate ZO-1 and Occludin Permeability: Reduce intestinal permeability (gallic acid reduces FITC glucan leakage)	Tight junction proteins: Upregulate occludin, ZO-1, claudin-1/3 Permeability: Reduce intestinal permeability Anti fibrosis: inhibits collagen synthesis Autophagy regulation: Increased expression of LC3B and Beclin-1; Downregulate Atg12 and Beclin-1	Tight junction protein: Restores ZO-1 expression and maintains TEER Permeability: Reduce intestinal permeability Epithelial protection: inhibits intestinal epithelial apoptosis; Activate FAK to enhance epithelial connectivity	Tight junction proteins: Upregulate occludin, ZO-1 and other proteins Permeability: Reduce intestinal permeability Cup shaped cells: promote proliferation and MUC2 secretion Epithelial protection: Inhibits epithelial cell apoptosis	Upregulation of tight junction protein and reduction of intestinal permeability

### 4.1 Flavonoids

Flavonoids exhibit anti-inflammatory effects by inhibiting inflammatory signaling pathways such as NF-κB, mitogen-activated protein kinase (MAPK, e.g., extracellular signal-regulated kinase [ERK]/C-Jun amino-terminal kinases [JNK]/p38), and activator protein-1 (AP-1). They reduce the release of pro-inflammatory cytokines including tumor necrosis factor-alpha (TNF-α), interleukin-1β (IL-1β), and IL-6. Additionally, flavonoids suppress the activity of cyclooxygenase-2 (COX-2), inducible nitric oxide synthase (iNOS), and myeloperoxidase (MPO), thereby blocking the amplification of the inflammatory cascade (Hu et al., 2022; Lyu et al., 2022; Al-Khayri et al., 2022; Xue et al., 2023).

In terms of antioxidant effects, flavonoids activate the Nrf2/antioxidant response element (ARE) pathway, upregulating the expression and activity of antioxidant substances such as heme oxygenase-1 (HO-1), superoxide dismutase (SOD), catalase (CAT), and glutathione (GSH), while reducing the levels of malondialdehyde (MDA), lipid peroxides (LPO), and excess nitric oxide (NO), thereby alleviating intestinal oxidative damage (Hu et al., 2022; Lyu et al., 2022; Al-Khayri et al., 2022; Liu et al., 2025).

In terms of gut microbiota modulation, it can optimize the microbial structure by increasing the abundance of beneficial bacteria such as *Lactobacillus*, Bifidobacterium, and Akkermansia muciniphila, while reducing the population of pathogenic bacteria such as *Enterococcus* and *Fusobacterium*. Additionally, it promotes the production of short-chain fatty acids (SCFAs) through microbial metabolism, thereby ameliorating IBD-associated dysbiosis (Hu et al., 2022; Lyu et al., 2022; Al-Khayri et al., 2022; Xue et al., 2023; Wang et al., 2021).

In terms of immunomodulation, flavonoids modulate the balance of T-cell subsets by promoting regulatory Treg differentiation and suppressing the polarization of Th1 and Th17 cells. Additionally, they inhibit macrophage activation toward the pro-inflammatory M1 phenotype, reduce neutrophil and dendritic cell infiltration, and enhance the secretion of the anti-inflammatory cytokine IL-10, thereby contributing to the maintenance of intestinal immune homeostasis (Al-Khayri et al., 2022; Xue et al., 2023; Liu et al., 2025; Louis-Jean, 2024).

Furthermore, it can protect the integrity of the intestinal mucosal barrier by upregulating the expression of intestinal tight junction proteins (ZO-1, Occludin, Claudin-1), reducing intestinal permeability, and decreasing bacterial translocation (Hu et al., 2022; Lyu et al., 2022; Xue et al., 2023; Wang et al., 2025). Certain flavonoids also regulate the intestinal endocrine system by promoting the secretion of gut hormones such as glucagon-like peptide-1 (GLP-1), glucagon-like peptide-2 (GLP-2), and cholecystokinin (CCK), thereby indirectly enhancing anti-inflammatory and intestinal repair effects (Li and Weigmann, 2022). Additionally, the intestinal targeting and bioavailability of flavonoids can be improved through formulation technologies such as nanocarriers and polymeric micelles (Lyu et al., 2022; Liu et al., 2025).

### 4.2 Phenolic acids

Phenolic acids exhibit anti-inflammatory effects by inhibiting the activation of NF-κB, MAPK (ERK/JNK/p38), and the NLRP3 inflammasome, downregulating the activity of inflammatory enzymes such as COX-2 and iNOS, and reducing the release of pro-inflammatory cytokines including TNF-α, IL-1β, and IL-6. For instance, ferulic acid derivatives C1/C1a decrease pro-inflammatory cytokine secretion in macrophages via suppression of NF-κB, while gallic acid blocks inflammation-induced intestinal epithelial barrier disruption by inhibiting myosin light chain kinase (MLCK) (Kim et al., 2024; Arinno et al., 2025).

In terms of antioxidant activity, it can activate the Nrf2/ARE pathway, upregulate the expression of antioxidant enzymes such as HO-1, SOD, CAT, and directly clear ROS and reduce MDA levels. For example, trans ferulic acid can clear free radicals such as OH and O _2_^-^ through the Nrf2 pathway, and the tannic acid metabolite urolith A (UA) can enhance antioxidant capacity through CYP1A1 activation (Zhang et al., 2025; Kujawska and Jodynis-Liebert, 2020).

In terms of regulating gut microbiota, phenolic acids can optimize the structure of the microbiota, increase the abundance of beneficial bacteria such as lactobacilli, bifidobacteria, Akkermansia, and reduce pathogenic bacteria such as Enterobacteriaceae and Desulfovibrio. Additionally, prebiotic components such as inulin can promote probiotic proliferation, such as catechins restoring the reduced Bacteroidetes abundance of dextran sodium sulfate (DSS)-induced ulcerative colitis via Nrf2/Slc7a11/Gpx4-dependent inhibition of ferroptosis signalling activation DSS and vanillic acid increasing the Firmicutes/Bacteroidetes ratio (Lu and Han, 2024; Harwansh et al., 2024).

In terms of immunomodulation, it can regulate the balance of T cell subsets (promoting Treg differentiation and suppressing Th1/Th17 polarization), inhibit macrophage polarization toward the M1 phenotype, and reduce neutrophil infiltration. For instance, ellagic acid (EA) balances the Th1/Th2 response, while ferulic acid decreases MPO activity to attenuate neutrophil migration (Kim et al., 2024; Kujawska and Jodynis-Liebert, 2020).

In terms of intestinal epithelial barrier protection, phenolic acids can repair the intestinal epithelial barrier and reduce intestinal permeability by upregulating tight junction proteins such as ZO-1 and Occludin (e.g., gallic acid reduces FITC-dextran leakage) (Arinno et al., 2025). Meanwhile, the use of nanocarriers (e.g., liposomes, polymeric micelles) can enhance the bioavailability and intestinal targeting of phenolic acid compounds (Harwansh et al., 2024).

### 4.3 Stilbenes

Stilbenoid compounds exert anti-inflammatory effects through multiple mechanisms. They inhibit the NF-κB signaling pathway by downregulating p65 transcriptional activity and NEMO ubiquitination (e.g., resveratrol), and by blocking NF-κB nuclear translocation (e.g., pterostilbene) (Shi et al., 2017; Zhang et al., 2024). Additionally, they modulate the MAPK and JAK/STAT pathways through suppression of ERK and JNK phosphorylation, as well as STAT1 activation, thereby reducing the release of pro-inflammatory cytokines such as TNF-α and IL-1β (Shi et al., 2017; Nunes et al., 2018; Gowd et al., 2022; Vaghari-Tabari et al., 2024). Furthermore, stilbenoids downregulate COX-2 and iNOS expression, leading to decreased production of prostaglandin E2 (PGE2) and NO (Shi et al., 2017; Vaghari-Tabari et al., 2024). They also regulate the Treg/Th17 balance via the mechanistic target of rapamycin (mTOR)–hypoxia-inducible factor α (HIF-1α) or IL-6–STAT3–HIF-1α signaling pathways (Shi et al., 2017; Gowd et al., 2022).

In terms of antioxidant activity, resveratrol can activate the Nrf2 Keap1 pathway to induce the expression of HO-1 and GCLC, increase SOD and glutathione peroxidase (GPx) activity, and reduce MDA levels (Nunes et al., 2018; Wellington et al., 2021; Hu et al., 2019). Pterostilbene has better ROS scavenging ability, and resveratrol can also enhance antioxidant stress resistance through the PPAR - γ pathway (Shi et al., 2017; Zhong et al., 2023).

In terms of gut microbiota modulation, resveratrol increases the abundance of *Lactobacillus*, Akkermansia, and SCFA-producing bacteria, while reducing harmful bacteria such as *Enterococcus faecalis* (Gowd et al., 2022; Vaghari-Tabari et al., 2024; Hu et al., 2019). Resveratrol exhibits extremely low oral bioavailability and primarily relies on specific gut microbiota for metabolic transformation. Among these, Slackia equolifaciens, Adlercreutzia equolifaciens, Eubacterium lenta ATCC 43055, and *Bacteroides* uniformis ATCC 8492 convert resveratrol into dihydroresveratrol (DH-RES) via hydrogenation, whereas *Bacillus* cereus produces piceid through glycosylation (Hu et al., 2019; Zheng et al., 2024). These metabolites not only significantly enhance the aqueous solubility and stability of resveratrol but also preserve or even augment its bioactivity (Hu et al., 2019; Zheng et al., 2024). For instance, DH-RES reduces pro-inflammatory cytokine levels by inhibiting the NF-κB pathway and exhibits superior ROS scavenging efficiency compared to vitamin E (Zheng et al., 2024). Polydatin helps maintain the expression of tight junction proteins in the intestinal epithelium and activates the Nrf2 antioxidant pathway. These metabolites thus serve as key functional forms through which resveratrol exerts intestinal protective effects (Zheng et al., 2024). Moreover, Polydatin, as a core microbial metabolite of resveratrol, can reciprocally modulate the composition of the gut microbiota by reducing the abundance of the pathogenic bacterium *E. faecalis* and increasing the populations of beneficial bacteria such as *Lactobacillus* and Bifidobacterium, thereby establishing a bidirectional “microbiota–polyphenol” interaction cycle (Zheng et al., 2024). Additionally, high concentrations of resveratrol (≥50 μM *ex vivo*, ≥100 mg/kg *in vivo*) inhibit sulfotransferase enzymes secreted by the gut microbiota, thereby blocking the conversion of resveratrol into less active sulfate esters (e.g., resveratrol-3-O-sulfate). This shifts more resveratrol toward active metabolic pathways (hydrogenation and glycosylation), further increasing the local intestinal concentrations of free resveratrol, DH-RES, and piceid (Shi et al., 2017; Hu et al., 2019; Zheng et al., 2024).

In terms of immunomodulation, these compounds can induce myeloid-derived suppressor cells (MDSCs) to suppress effector T cells, promote M2 macrophage polarization, inhibit Th1 and Th17 cells, and induce Tregs (Shi et al., 2017; Nunes et al., 2018; Gowd et al., 2022; Vaghari-Tabari et al., 2024; Wellington et al., 2021; Hu et al., 2019). Additionally, they can modulate immune responses by upregulating miR-101b and downregulating miR-31 (Shi et al., 2017; Gowd et al., 2022).

In addition, resveratrol improves the intestinal epithelial barrier by upregulating tight junction protein expression, inhibits collagen synthesis to exert anti-fibrotic effects, and protects the intestinal mucosa by modulating autophagy (Shi et al., 2017; Gowd et al., 2022; Wellington et al., 2021; Hu et al., 2019). Pterostilbene, due to its methylation modification, exhibits higher bioavailability and metabolic stability (Zhang et al., 2024; Zhong et al., 2023; Chen et al., 2024).

### 4.4 Lignans

In terms of anti-inflammatory effects, flax linoorbitides (LOBs) and enterolactone (ENL) downregulate TNF-α and upregulate PPAR-γ expression to inhibit inflammatory signaling (Almousa et al., 2018). SDG and its metabolites ENL/enterodiol (END) reduce the release of IL-1β and IL-6 by suppressing the NF-κB pathway (Plaha et al., 2022). Moreover, SDG and sesamin synergistically inhibit lipoxygenase (LOX)/COX-2 activity. Sesamin suppresses COX-2/LOX activity, thereby reducing inflammatory mediators, while also downregulating NF-κB nuclear translocation (Bai et al., 2019). Schisandrin B from Schisandra chinensis decreases pro-inflammatory cytokines and reduces neutrophil infiltration through inhibition of the MAPK/NF-κB pathway (Ma et al., 2021).

In terms of antioxidant effects, ENL and LOBs enhance SOD/CAT activity and reduce MDA levels (Almousa et al., 2018); SDG protects the intestinal mucosa through free radical scavenging capacity and upregulation of antioxidant enzymes (Plaha et al., 2022); flaxseed lignans also activate the Nrf2 pathway to upregulate HO-1 expression (Merakeb et al., 2025); and sesamin elevates GSH levels to enhance reducing capacity (Bai et al., 2019).

Regarding the regulation of gut microbiota, secoisolariciresinol diglucoside (SDG) must be converted by bacteria such as *Bacteroides* and *Clostridium* into ENL and END (Plaha et al., 2022). Concurrently, lignans can enrich SCFA-producing bacteria (e.g., Ruminococcus) and inhibit pathogenic bacteria (e.g., *Clostridium* sensu stricto-1), thereby restoring microbial homeostasis (Baldi et al., 2023; Caban et al., 2025). Sesamin has been shown to increase the abundance of beneficial bacteria such as Bifidobacterium (Bai et al., 2019).

In terms of immunomodulation, Schisandrin B targets the STAT3 signaling pathway to inhibit Th17 cell differentiation and interleukin-17A (IL-17A) production (Ma et al., 2021). Flaxseed lignans suppress Th17 cell differentiation and promote the proliferation of Tregs, thereby modulating mucosal immunity (Merakeb et al., 2025). Additionally, ENL reduces immune infiltration by restoring the Th17/Treg balance (Baldi et al., 2023). Lignin can also affect the inflammatory activation status of immune cells such as macrophages, reduce neutrophil infiltration, and decrease intestinal damage mediated by B cell autoantibodies (Almousa et al., 2018; Baldi et al., 2023).

With respect to intestinal epithelial barrier protection, ENL has been demonstrated to restore the expression of zonula occludens-1 (ZO-1) and maintain trans-epithelial electrical resistance (TEER) (Almousa et al., 2018). Flaxseed lignans inhibit intestinal epithelial apoptosis and activate the focal adhesion kinase (FAK) pathway (Merakeb et al., 2025). Furthermore, Schisandrin B enhances epithelial junction integrity through FAK activation (Ma et al., 2021).

### 4.5 Coumarins

In terms of anti-inflammatory effects, coumarin-related compounds primarily exert their functions by inhibiting the NF-κB and MAPK signaling pathways (Ju et al., 2024; Di Stasi, 2021). They either downregulate IKKα/β phosphorylation to prevent IκBα degradation or inhibit NF-κB p65 nuclear translocation, thereby reducing the expression of pro-inflammatory cytokines such as TNF-α and IL-1β (Ju et al., 2024; Di Stasi, 2021). Concurrently, these compounds can suppress the activities of COX-2 and 5-LOX, leading to decreased production of inflammatory mediators including PGE_2_ and LTB_4_ (Di Stasi, 2021; Rostom et al., 2022). Some derivatives are also capable of blocking the JAK/STAT3 pathway or inhibiting NLRP3 inflammasome activation (Witaicenis et al., 2018).

In terms of antioxidant properties, the metabolites of psoralen and its isomers converted by gut microbiota alleviate H_2_O_2_ - induced oxidative stress activation in intestinal cells, which is superior to the prototype (Liu et al., 2019). Aesculin and esculetin activate the Nrf2 pathway, promote the expression of SOD and GPx, and reduce ROS and MDA levels (Ju et al., 2024). Isocoumarin antagonizes GSH depletion and inhibits MPO (Di Stasi, 2021). 4-Methylesculetin upregulates HO-1 (Di Stasi, 2021). Urolithin A/B, metabolites derived from gut microbiota, elevate GSH via the Nrf2 pathway (Di Stasi, 2023). Bergapten enhances Nrf2 phosphorylation through the PI3K/Akt pathway (Witaicenis et al., 2014). Moreover, the combination of esculetin and osthole at a 1:1 ratio synergistically activates the Nrf2 signaling pathway (Rostom et al., 2022).

In the regulatory role of gut microbiota, on one hand, intestinal flora can convert prototype coumarins into more active metabolites (e.g., 7-methoxycoumarin is converted into coumaric acid) (Witaicenis et al., 2018; Liu et al., 2019); on the other hand, related compounds can increase the abundance of beneficial bacteria such as *Lactobacillus*, Bifidobacterium, SCFA-producing bacteria (e.g., Faecalibacterium prausnitzii), and Akkermansia muciniphila, while inhibiting the biofilm formation of pathogenic bacteria such as *Escherichia coli* (Di Stasi, 2021; Rostom et al., 2022; Witaicenis et al., 2014).

In terms of immunomodulatory effects, coumarins can regulate the Treg/Th17 balance and enhance the immunosuppressive function of Treg cells (Di Stasi, 2021). They promote macrophage polarization toward the M2 phenotype, leading to the secretion of IL-10 and TGF-β, while inhibiting the pro-inflammatory response of M1 macrophages (Witaicenis et al., 2018). Additionally, coumarins reduce Th1 cell infiltration and facilitate the differentiation of tolerogenic DCs, thereby diminishing DC-mediated T cell activation (Rostom et al., 2022; Witaicenis et al., 2014). Through these multi-cellular regulatory mechanisms, they enhance intestinal immune tolerance. Furthermore, coumarins modulate B lymphocyte function, reduce abnormal secretion of immunoglobulin A (IgA) in the intestinal mucosa, and prevent excessive activation of intestinal immunity (Witaicenis et al., 2018).

In terms of intestinal epithelial barrier protection, coumarins upregulate the expression of tight junction proteins such as occludin and ZO-1, as well as E-cadherin, thereby reducing intestinal epithelial permeability (Di Stasi, 2021; Witaicenis et al., 2018). Additionally, they promote goblet cell proliferation and MUC2 secretion, leading to thickening of the mucus layer. Concurrently, coumarins inhibit intestinal epithelial cell apoptosis, thereby preserving epithelial integrity (Rostom et al., 2022; Witaicenis et al., 2018).

## 5 Polyphenols in animal models and clinical research progress

### 5.1 Animal research

In recent years, animal experiments have actively explored the potential application of polyphenols in the treatment of CD and have made some remarkable progress (Table 2). In animal model research, polyphenols have been shown to potentially alleviate intestinal inflammation. First, the study conducted by Maria *et al.* with the use of pomegranate extract (PE) rich in EA in a 2,4,6-trinitrobenzenesulfonic acid (TNBS)-induced rat model of CD is fascinating. This highlights the multitude of pathways that polyphenols can influence. The reductions in MPO activity, TNF-α concentration, and the expression of COX-2 and iNOS indicate the anti-inflammatory potential of PE. The fact that PE also inhibits the phosphorylation of MAPKs and the nuclear translocation of NF-κB underscores the multifaceted nature of the influence of polyphenols on cellular signaling pathways (Rosillo et al., 2012). Second, tannic acid, another polyphenol, not only has the potential to reduce inflammation but also promotes mucus production in colonic goblet cells. The latter is crucial because mucus serves as a protective barrier in the gut, preventing direct contact between gut microbes and the intestinal epithelium and thus maintaining gut homeostasis (Rosillo et al., 2011).

**TABLE 2:** Relevant studies on polyphenols in animal experiments.

Serial number	Polyphenol	CD model	Dosage/Duration of intake	Effects	References
1	PE and EA	Trinitrobenzensulfonic acid (TNBS); Four-week-old male Wistar rats	Rats were fed with different diets during 30 days before TNBS instillation and 2 weeks before killing: (i) PE 250 mg/kg/day, (ii) PE 500 mg/kg/day (iii) EA 10 mg/kg/day (iv) EA 10 mg/kg/day + PE 250 mg/kg/day	(1) PE and an EA-enriched PE diets drastically decreased COX-2 and iNOS overexpression (2) Reduced MAPKs phosporylation (3) Prevented the nuclear Nf-kB translocation	Rosillo et al. (2012)
2	Resveratrol	Peptidoglycan-polysaccharide (PGPS); Specific pathogen free (SPF) female Lewis rats, 8–10 weeks of age and weighing approximately 150–165 g	20 mg/kg/d, 40 mg/kg/d, and 100 mg/kg/d; resveratrol dosing; 27days	(1) Resveratrol decreased cecal wall fibrosis as assessed histologically (2) Reduced inflammatory cytokines (IL-1, IL-6, and TNF-α) and profibrotic factors such as TGF-β1 (3) Downregulation of insulin-like growth factor 1 (IGF-1) and procollagen mRNA expression	Rahal et al. (2012)
3	Polyphenolic maqui extract (Ach)	TNBS; Male BALB/c mice aged 12–14 weeks old, weighing 19–35 g	50 g/kg/day; 4 days, 11day	(1) Ach administration inhibited body weight loss and colon shortening, and attenuated the macroscopic and microscopic damage signs, as well as significantly reducing transmural inflammation and boosting the recovery of the mucosal architecture and its muco‐secretory function (2) Ach promotes macrophage polarization to the M2 phenotype (3) Down‐regulating significantly the expression of inflammatory proteins COX‐2 and iNOS (4) It regulates the antioxidant Nrf‐2/HO‐1 pathway	Ortiz et al. (2020)
4	EA	TNBS; Male Wistar rats, weighing 180–220 g	10–20 g/kg; 4 times	(1) EA increased mucus production in goblet cells in colon mucosa (2) Decreased neutrophil infiltration and pro-inflammatory proteins COX-2 and iNOS overexpression (3) EA was capable of reducing the activation of p38, JNK and ERK1/2 MAPKs (4) Preventing the inhibitory protein IkB degradation (5) Inducing an inhibition of the nuclear translocation level of p65 in colonic mucosa	Rosillo et al. (2011)
5	green tea polyphenols (GrTP, EGCG)	IL-10 deficient mice (BALB/c-background)	1%, 0.5%, 0.25% GrTP; 10 days, 10 weeks	(1) Reduced the loss in body weight (2) Improved anemia and the hematocrit value (3) Improved enterocolitic symptoms while (4) Reduced secretion of IL-6 levels (5) Improved intestinal GSH.	Oz et al. (2013)
6	Olea europaea leaf extract	DNBS; Male CD1 mice (8–10 weeks of age) DSS; Male C57BL/6J mice (7–9 weeks old)	1, 10 and 25 mg/kg/day; 6 days 0.5, 1 and 10 mg/kg, 11 days	(1) Reducing the expression of pro-inflammatory mediators (IL-1β, TNF-α and iNOS) (2) Improving the intestinal epithelial barrier integrity (3) Restoring the expression of ZO-1, MUC-2 and TFF-3	Vezza et al. (2017)
7	Rutin and quercetin	TNBS; Female Wistar rats (175–225 g)	Rutin and quercetin were administered in equimolar doses (20 and 11.1 mg/kg, respectively, equivalent to 32.8 μmol/kg body weight)	(1) Reduction in body weight loss and alleviation of anorexia symptoms (2) The ileal injury score, weight-to-length ratio, MPO activity, and AP activity were all markedly lowered (3) The elevated mRNA levels of IL-1β and IL-17 were normalized	Mascaraque et al. (2015)
8	Anthocyanin-Rich Extract	SAMP mouse	53 mg/kg/day; 10 weeks	(1) It reduced the levels of pro-inflammatory bacteria (such as *Bacteroides* and ASF356) and promoted the growth of potentially beneficial bacteria (including Lachnospiraceae and Parabacteroides) (2) It mitigated body weight loss and attenuated ileal inflammation, demonstrated by reduced inflammatory scores, less immune cell infiltration, and decreased necrosis (3) It inhibited the development of colonic tertiary lymphoid organs	Verna et al. (2025)
9	Luem Pua rice extract	Indomethacin; Male Wistar rats (6–8 weeks old, the average weight of 250 g)	5 g/kg/day; 7 days	(1) It did not significantly alter the abundance of butyrate-producing bacteria (e.g., Lachnospiraceae NK4A136 group and Coprococcus 2), and showed no significant suppressive effect on potential pathogens (such as *Bacteroides* and *Fusobacterium*) (2) It significantly increased the abundance of the phylum Proteobacteria	Thipart et al. (2023)
10	Grape seed extract	IL-10-deficient female mice	1%; 16 weeks	(1) Significantly reduce crypt depth, increase the villus/crypt ratio, and optimize ileal mucosal structure; (2) Increase goblet cell density and enhance epithelial cell differentiation capacity; (3) Decrease the phosphorylation level of NF-κB p65; (4) Restore abnormally elevated Beclin-1 and phosphorylated AMPK to normal levels; (5) Upregulate the barrier-forming tight junction protein Claudin-1, downregulate the pore-forming tight junction protein Claudin-2, reduce epithelial barrier leakage, and enhance ileal barrier function	Yang et al. (2014)
11	copper ion–luteolin nanocomplexes	TNBS	10 mg/kg, 20 mg/kg; 5 days	(1) Reduce body weight loss and mortality rate, and restore colon length; (2) Repair colonic mucosal damage and reduce inflammatory cell infiltration; (3) Simultaneously decrease MDA and MPO levels, while restoring SOD and CAT activity; (4) Reduce pro-inflammatory factors such as TNF-α and IL-1β, upregulate anti-inflammatory factors such as IL-4 and IL-10, and suppress the expression of intestinal fibrosis-related TGF-β; (5) Increase goblet cell count and mucus secretion, and repair the intestinal epithelial barrier	Fu et al. (2025)
12	Linseed	CEAABAC10 transgenic mice fed high-fat diet and challenged with AIEC LF82 pathogen	Each 100g of this group’s diet contains 1.01g of flaxseed oil; Each 100g of this group’s diet contains 6g of squeezed flaxseed; 12 weeks	(1) Reduce fecal lipocalin-2 levels and suppress intestinal inflammation; (2) Increase the Firmicutes/Bacteroidetes ratio, and significantly elevate the abundance of Clostridiales, Prevotella, Paraprevotella, and Ruminococcus	Plissonneau et al. (2022)

Furthermore, polyphenols, such as maqui extract (Ach), can influence the redox state of cells. By regulating the nuclear erythroid 2-related factor 2/hemoxygenase-1 (Nrf-2/HO-1) pathway, these polyphenols can enhance the antioxidant capacity of cells, further mitigating inflammation and tissue damage. Additionally, promoting the differentiation of macrophages toward the M2 phenotype is another beneficial anti-inflammatory action since M2 macrophages are generally associated with tissue repair and wound healing (Ortiz et al., 2020). Resveratrol, found in red wine and grapes, is a potent antioxidant that has been shown to reduce fibrosis in CD models. Fibrosis, or the thickening and scarring of connective tissue, is a common complication of CD and can lead to obstruction of the intestines. By reducing fibrosis, resveratrol may help prevent these obstructions and maintain intestinal function (Rahal et al., 2012). (−)-epigallocatechin-3-gallate (EGCG), or epigallocatechin gallate, is a type of catechin found in green tea that has demonstrated beneficial effects on improving colitis in CD models. Colitis, or inflammation of the colon, is a prominent feature of CD, and by reducing inflammation, EGCG can potentially alleviate some of the symptoms of CD, such as abdominal pain and diarrhea (Oz et al., 2013). Olive leaf extract, rich in polyphenols, has been shown to improve the integrity of the intestinal epithelial barrier. The intestinal epithelium serves as the first line of defense against harmful pathogens and substances in the gut. By maintaining the integrity of this barrier, olive leaf extract can potentially prevent the triggering of an immune response that leads to inflammation in patients with CD. In addition, it also reduces the expression of proinflammatory mediators such as IL-1β, TNF-α, and iNOS, further aiding in the management of inflammation in CD (Vezza et al., 2017).

In studies on other polyphenols, oral administration of rutin (the glycosylated form of quercetin) has demonstrated significant anti-inflammatory effects in both ileitis and colitis models, whereas its aglycone (quercetin) exhibited weaker efficacy (Mascaraque et al., 2015). A diet supplemented with anthocyanin-rich extracts may serve as a potential adjuvant intervention for CD by modulating the gut microbiota and directly acting on host tissues to alleviate inflammation (Verna et al., 2025). Grape seed extract appears to protect the ileal epithelium in IL10KO mice by inhibiting inflammatory responses (e.g., downregulating the NF-κB pathway) and modulating autophagy (e.g., downregulating the AMPK–Beclin-1 pathway), thereby restoring normal proliferation and differentiation of epithelial cells (Yang et al., 2014). Copper–luteolin nanocomplexes have also shown beneficial effects, significantly reducing mortality in a CD model (from 37.5% to 12.5%), repairing the colonic mucosa, and suppressing intestinal fibrosis (Fu et al., 2025). Furthermore, flaxseed was found to suppress gut inflammation associated with CD, and extruded flaxseed—due to its matrix components such as fiber and lignans—additionally modulated the mucosa-associated microbiota and promoted butyrate production (Plissonneau et al., 2022). However, not all extracts exhibited significant efficacy; for instance, deep purple rice extract showed no notable beneficial regulatory effects in a chronic CD model (Thipart et al., 2023).

The above research results indicate that the intake of polyphenols is significantly associated with a reduction in the symptoms of CD in animals, such as reduced swelling and ulcer area, decreased levels of inflammatory markers, and improved structural integrity of the intestinal mucosa. Although the promising results derived from animal model studies are noteworthy, it is crucial to acknowledge that these outcomes may not necessarily be extrapolated seamlessly to humans due to inherent biological differences. Consequently, researchers have pursued clinical trials to further validate these findings in a human context.

### 5.2 Clinical research

With the gradual increase in clinical research, empirical evidence for the potential role of polyphenols in the treatment of CD is gradually accumulating (Table 3). In several small-scale trials, polyphenols from various sources, such as green tea polyphenols, olive leaf extract polyphenols, and other natural plant-based polyphenols, have been assessed to determine their effects on the incidence rate, clinical symptoms, and quality of life in patients with CD (Kolacek et al., 2019; Lu et al., 2017; Koláček et al., 2013; Suskind et al., 2013; Holt et al., 2005; Sugimoto et al., 2020; Swanson et al., 2011; Kim et al., 2020; Kim et al., 2021; Ng et al., 2015; Papada et al., 2018).

**TABLE 3:** Relevant studies on polyphenols in clinical practice.

Serial number	Polyphenol	Subject description	Study type	Dosage/Duration of intake	Effects	NC number	References
1	Polyphenolic extract Pycnogenol	14 children suffering from CD	non-randomized controlled trial	2 mg/kg/day; 10 weeks	(1) Decreased the level of the dynamic form of thromboxane B2	-	Kolacek et al. (2019)
2	Dietary Polyphenols	110 incident cases of CD (73% women; mean age at diagnosis = 55.4 years, SD 11.1)	Prospective cohort study	Total polyphenols, daily median intakes 1,091.6 mg/d, (848.8–1,517.4) Resveratrol, daily median intakes 0.1 mg/d, (0.0–0.2)	(1) Total polyphenol intake was not associated with CD (2) Resveratrol was an inverse association with CD.	-	Lu et al. (2017)
3	Pycnogenol® (Pyc)	15 pediatric patients diagnosed with CD (7 males and 8 females; age, 13–18 years)	Non-randomized controlled trial	2 mg/kg/day, 10 weeks	(1) Pyc significantly increased the levels of DAO, SOD and GPX activities in patients with CD after 5 and 10 weeks of its administration (2) After 10 weeks of administration Pyc caused reduction in serum iron concentration, which resulted in an increase in sTfR levels	NCT01426568	Koláček et al. (2013)
4	Curcumin	6 with CD	Non-randomized controlled trial	Initially, received 500 mg twice a day for 3 weeks, Using the forced dose titration design, doses were increased to 1 g twice daily at week 3 for a total of 3 weeks and then titrated again to 2 g twice daily at week 6 for 3 weeks	(1) PCDAI dropped from 5 to 0 suggesting improvement	NCT00889161	Suskind et al. (2013)
5	Curcumin	5 with CD	Non-randomized controlled trial	360 mg three times daily for 1 month and then 360 mg four times daily for the remaining 2 months	(1) The CDAI scores for all completed subjects fell, with a mean reduction of 55 points; sedimentation rate fell as well, with a mean reduction of 10 mm/h. CRP was reduced by a mean of 0.1 mg/dL	-	Holt et al. (2005)
6	Curcumin derivative Theracurmin®	17 men and women with mil-to-moderate CD	Randomized, Double-Blind, Multicenter Study	360 mg/days; 12 weeks	(1) Reduction in CDAI, simple endoscopic score for Crohn’s disease (SESCD) stool frequency and abdominal pain (2) Reduction in anal lesions	-	Sugimoto et al. (2020)
7	Red wine	6 with inactive CD	Non-randomized controlled trial	1–3 glasses of red wine a day for 1 week (approx. 0.4 g EtOH/kg)	(1) Decreased stool CRP (2) Increased intestinal permeability	-	Swanson et al. (2011)
8	Mango	3 with CD	Non-randomized controlled trial	200–400 g mango pulp/8 weeks	(1) Improved the primary outcome SCCAI score (2) Decreased the plasma levels of pro-inflammatory cytokines including IL-8, growth-regulated oncogene (GRO) and GM-CSF by 16.2%, 25.0% and 28.6% (3) Increasing the abundance of *Lactobacillus* spp., *Lactobacillus* plantarum, *Lactobacillus* reuteri and *Lactobacillus* lactis (4) Increased fecal butyric acid production	NCT02227602	Kim et al. (2021)
9	Daily tea or coffee consumption	186 with CD	Case control study	International organization of IBD Environmental factor questionnaire—food habits (daily tea (aOR 0.63; 0.46–0.86) or coffee consumption (aOR 0.51; 0.36–0.72))	(1) Tea reduced odds of CD.	-	Ng et al. (2015)
10	Pistacia lentiscus (mastiha)	40 with CD	Randomised, Double-Blind, Placebo-Controlled Trial	Natural PL supplement at a dose of 2.8 g daily (four tabs × 700 mg PL); 3 months	(1) Decreased The levels of low-density lipoprotein (oxLDL)/LDL, thioproline and lysine (2) Ameliorated a decrease in plasma-free AAs	NCT02796339	Papada et al. (2018)

Clinical research reveals the potential ameliorative effects of specific polyphenols on patients with CD, involving mechanisms such as anti-inflammation, antioxidation, and metabolic regulation. One study showed that after 10 weeks of treatment with a polyphenol alcohol extract, 14 pediatric CD patients experienced a significant decrease in blood thromboxane levels—a key factor promoting platelet aggregation and vasoconstriction—which may help reduce inflammation and improve gut health (Kolacek et al., 2019; Hawkey et al., 1983). Resveratrol intake or status exhibits a negative correlation with CD, suggesting it may reduce CD incidence or severity (Lu et al., 2017).

Research in CD patients in remission found that Pycnogenol® (French maritime pine bark extract), while not significantly affecting conventional inflammatory markers or disease activity indices, improved iron metabolism markers (increased transferrin, decreased ferritin), indicating a potential role in regulating the frequently disrupted iron homeostasis in CD. Concurrently, it effectively reduced oxidative stress by enhancing antioxidant enzyme (SOD, GPX) activity and lowering levels of lipid (LOP, eight-isoP) and protein (advanced oxidation protein products [AOPP]) oxidative damage markers. It may also mitigate histamine-induced inflammation by increasing diamine oxidase (DAO) levels, an enzyme crucial for detoxifying the inflammatory mediator histamine (Koláček et al., 2013). Curcumin demonstrated positive effects in clinical studies, significantly reducing the pediatric crohn’s disease activity index (PCDAI) or crohn’s disease activity index (CDAI) scores and erythrocyte sedimentation rate (ESR) in CD patients (Suskind et al., 2013; Holt et al., 2005). Its highly absorbable derivative, Theracurmin®, further improved stool frequency, abdominal pain, and perianal lesions in a randomized double-blind study (Sugimoto et al., 2020). Moderate red wine consumption for 1 week was found to reduce fecal CRP and improve intestinal permeability. However, non-active CD patients drinking red wine daily may face an increased long-term risk of relapse. As the study could not distinguish whether alcohol or other components in red wine (e.g., polyphenols) were responsible, these findings require cautious interpretation and warrant further investigation (Swanson et al., 2011). Mango intake significantly improved patients’ simple clinical colitis activity index (SCCAI) scores, reduced levels of pro-inflammatory cytokines (e.g., interleukin-8 [IL-8], GRO, granulocyte macrophage colony-stimulating factor [GM-CSF]), and beneficially altered the gut microbiota (increased *Lactobacillus* abundance and butyrate levels) (Kim et al., 2020; Kim et al., 2021). Epidemiological studies suggest daily tea or coffee consumption may be associated with a reduced CD risk, though excessive intake may cause gastrointestinal discomfort (Ng et al., 2015). Additionally, Pistacia lentiscus extract has been reported to improve oxidative stress in CD patients by ameliorating plasma amino acids (AAs) profiles and reducing oxidative damage markers (e.g., oxidized low-density lipoprotein/low-density lipoprotein [oxLDL/LDL]) (Papada et al., 2018).

While these studies suggest polyphenol interventions may improve surrogate markers in CD—such as reducing thromboxane, altering iron metabolism/oxidative stress markers, lowering clinical activity indices, modulating cytokines/microbiota, or showing epidemiological associations—the evidence remains inconclusive due to significant limitations. Crucially, these findings stem primarily from small, heterogeneous studies using non-standardized extracts/doses, lack robust evidence, and focus on biochemical or symptomatic surrogates rather than clinically relevant hard endpoints, such as endoscopic remission, reduced hospitalizations, or avoidance of surgery. Importantly, studies like the one on Pycnogenol® highlight inconsistencies, showing no effect on established inflammatory biomarkers or disease activity scores despite improvements in iron metabolism. Concurrently, the potential increased relapse risk in inactive CD patients from daily red wine consumption and the gastrointestinal discomfort from tea/coffee introduce uncertainty regarding the clinical significance of polyphenol therapy. Although there is currently a lack of clinical studies on resveratrol intervention specifically targeting patients with CD, a randomized controlled trial conducted in patients with ulcerative colitis (UC) offers valuable insights. The results of this trial demonstrated that resveratrol intervention significantly reduced TNF-α and hs-CRP levels, suppressed NF-κB activity, and improved patients’ quality of life and clinical symptoms (Samsami-Kor et al., 2015). Although there are differences in disease types between UC and CD, these findings undoubtedly provide preliminary clinical evidence for the anti-inflammatory potential of resveratrol. However, there are still limitations, and future research should conduct resveratrol intervention trials to further validate the therapeutic effects on CD patients. Large-scale randomized controlled trials employing standardized polyphenol preparations and prioritizing objective indicators of mucosal healing and clinical outcomes are essential to definitively establish the therapeutic role of polyphenols in CD management.

## 6 Challenges and prospects

### 6.1 The complex interaction between bioavailability, metabolism, and intestinal pathology

The clinical application of polyphenols is severely limited by their inherent characteristics, including chemical instability, low absorption rate in the gastrointestinal tract, and extensive and rapid metabolism by host enzymes and gut microbiota, resulting in generally low bioavailability of most polyphenols and significant individual differences. This is mainly influenced by various factors such as its different phenolic components, food matrix, host genetics, and gut microbiome (El-Saadony et al., 2024; Galmés et al., 2021; Scott et al., 2022). Most polyphenols undergo hydrolysis in the small and large intestine, promoting their absorption and reducing their potential toxicity. Subsequently, polyphenols enter the colon and undergo coupling reactions upon uptake into intestinal epithelial cells. The absorption of glycoside form polyphenols by the stomach, small intestine, and large intestine (accounting for approximately 5%–10% of the total polyphenol intake) depends on factors such as hydrophobicity or lipophilicity. Almost all polyphenols absorbed in the intestine are transported through the portal vein and undergo phase II metabolism in the liver, forming conjugated polyphenol metabolites that are then transported back to the gastrointestinal tract for further metabolism and/or excreted in the form of feces (Scott et al., 2022). However, this metabolic process may be significantly altered in Crohn’s disease patients. Intestinal inflammatory environment can lead to impaired intestinal epithelial barrier function and disrupted gut microbiota (Sharma et al., 2025). This pathological state is like a double-edged sword: on the one hand, it may increase the passive penetration of polyphenols through the damaged mucosa, enhancing its local exposure; On the other hand, it may alter the contact time between polyphenols and their target of action, ultimately leading to unpredictable bioavailability and therapeutic efficacy (Langeraert et al., 2025). Therefore, when evaluating the efficacy of polyphenols in CD, individual differences in their absorption and metabolism must be fully considered.

### 6.2 Dose dependent dual roles: from antioxidant to prooxidant

Polyphenols exhibit dual effects of antioxidant and prooxidant at different concentrations. At low doses in physiology, polyphenols mainly act as antioxidants by scavenging ROS and chelating transition metal ions to inhibit the Fenton reaction and exert antioxidant effects (Dzah et al., 2024; Rudrapal et al., 2022; Andrés et al., 2023). However, under high doses or specific conditions (such as high pH values and the presence of transition metal ions), polyphenols may transform into prooxidants (León-González et al., 2015; Ouyang et al., 2020). The mechanism of promoting oxidation includes: generating ROS such as H_2_O_2_ through self-oxidation; Alternatively, under the catalysis of metal ions (especially copper ions with high concentrations in tumor tissue), highly active hydroxyl radicals can be generated through Fenton or Fenton like reactions, thereby inducing DNA damage and cell apoptosis (León-González et al., 2015; D'Angelo et al., 2017). However, this potent prooxidant effect may also lead to adverse reactions. Studies have reported that treatment of freshly isolated rat hepatocytes with 200 μM EGCG for 24 h resulted in a dose-dependent reduction in liver function and disruption of mitochondrial membrane potential (Ouyang et al., 2020). Additionally, when mouse blastocysts were exposed to 25–50 μM EGCG, increased apoptosis and decreased cell numbers were observed, indicating impaired embryonic development (Ouyang et al., 2020). In human lymphocytes from healthy subjects, EGCG at concentrations of 1–100 μM induced dose-dependent DNA strand breaks (though no clastogenic effects were observed *in vivo*) (Ouyang et al., 2020). These findings suggest that high-dose EGCG supplementation may cause hepatotoxicity, nephrotoxicity, and DNA damage in lymphocytes. Therefore, the potential risks of high-dose polyphenol supplementation cannot be ignored and must be rigorously evaluated in future dose exploration studies.

### 6.3 Future outlook

Overcoming the aforementioned challenges is crucial for advancing polyphenols toward clinical application. Future research should focus on the following directions: First, efforts should be directed toward developing novel delivery systems (e.g., nanocarriers, microemulsions) and combination therapy strategies to enhance the bioavailability and stability of polyphenols. Second, standardized, chemically well-characterized plant extracts or purified compounds should be employed to ensure the comparability and reproducibility of research outcomes. Third, in-depth investigation into pharmacokinetics and safety profiles is essential. Studies should aim to elucidate the *in vivo* processes of these compounds, including the mechanisms underlying their low bioavailability and inter-individual variability. Systematic evaluation of their dose-dependent pro-oxidant effects and potential hepatorenal toxicity is necessary to determine the safety window and provide a scientific basis for clinical dosing. Fourth, large-scale randomized controlled trials are required to generate robust evidence-based medical data regarding the clinical efficacy and long-term safety of these compounds, which is pivotal for their translation into clinical practice. By advancing these research priorities, the clinical application pathway for polyphenols can be clarified and their therapeutic efficacy optimized.

## Glossary

CD: Crohn’s disease
IBD: Inflammatory bowel disease
IL-6: Interleukin-6
LPS: Lipopolysaccharides
TNF-α: Tumor necrosis factor-α
NF-κB: Nuclear factor kappa-B
NK: Natural killer
IFN-γ: Interferon-γ
CCR2: C-C Motif Chemokine Receptor 2
RANTES: Regulated on Activation, Normal T-Cell Expression and Secretion
DCs: Dendritic cells
IL-12: Interleukin-12
IL-18: Interleukin-18
IL-23: Interleukin-23
TGF-β: Transforming growth factor-β
IL-17: Interleukin-17
Tregs: Regulatory T cells
MAPK: Mitogen-activated protein kinase
ERK: Extracellular signal-regulated kinase
JNK: C-Jun amino-terminal kinases
COX-2: Cyclooxygenase-2
LOX: Lipoxygenase
ROS: Reactive oxygen species
SOD: Superoxide dismutase
CAT: Catalase
GSH: Glutathione
GPx: Glutathione peroxidase
SCFA: Short-chain fatty acid
IL-8: Interleukin-8
PGE2: Prostaglandin E2
iNOS: Inducible nitric oxide synthase
NO: Nitric oxide
TNBS: 2,4,6-trinitrobenzenesulfonic acid
PE: Pomegranate extract
EA: Ellagic acid
Ach: Polyphenolic maqui extract
Nrf-2/HO-1: Nuclear erythroid 2-related factor 2/hemoxygenase-1
CRP: Calprotectin
PCDAI: Pediatric Crohn’s Disease Activity Index
AOPP: Advanced oxidation protein products
DAO: Diamine oxidase
CDAI: Crohn’s disease activity index
SCCAI: Simple Clinical Colitis Activity Index
GRO: Growth-regulated oncogene
GM-CSF: Granulocyte macrophage colony-stimulating factor
oxLDL/LDL: Oxidized low-density lipoprotein/low-density lipoprotein
AAS: Amino acids
IL-1β: Interleukin-1β
NSAIDs: Non-steroidal anti-inflammatory drugs
Th17: T helper 17
Th1: T helper 1
IL-23R: IL-23 receptor
JAK: Janus kinase
STAT: Signal transducer and activator of transcription
Nrf2: Nuclear factor erythroid 2-related factor 2
AP-1: Activator protein-1
MPO: Myeloperoxidase
ARE: Nrf2/antioxidant response element
HO-1: Heme oxygenase-1
MDA: Malondialdehyde
LPO: Lipid peroxides
GLP-1: Glucagon-like peptide-1
GLP-2: Glucagon-like peptide-2
CCK: Cholecystokinin
MLCK: myosin light chain kinase
UA: Urolith A
DSS: Dextran sodium sulfate
mTOR: Mechanistic target of rapamycin
HIF-1α: Hypoxia-inducible factor α
DH-RES: Dihydroresveratrol
MDSC: Myeloid-derived suppressor cell
LOB: Linoorbitide
ENL: Enterolactone
END: Enterodiol
IL-17A: Interleukin-17A
ZO-1: Zonula occludens-1
TEER: Trans-epithelial electrical resistance
FAK: Focal adhesion kinase
IgA: Immunoglobulin A
UC: ulcerative colitis
EGCG: (−)-epigallocatechin-3-gallate
